# Multi‐Step and Switchable Energy Transfer in Photoluminescent Organosilicone Capsules

**DOI:** 10.1002/advs.202402565

**Published:** 2024-06-18

**Authors:** Longyue Yu, Hailong Liu, Ning Feng, Gang Yi, Xia Xin, Jingcheng Hao, Hongguang Li

**Affiliations:** ^1^ Key Laboratory of Colloid and Interface Chemistry Ministry of Education School of Chemistry and Chemical Engineering Shandong University Jinan Shandong 250100 China; ^2^ Shandong Key Laboratory of Advanced Organosilicon Materials and Technologies Zibo 256401 China; ^3^ National Engineering Research Center for Colloidal Materials Shandong University Jinan 250100 China

**Keywords:** light‐harvesting systems, overall energy transfer efficiency, sequential energy transfer

## Abstract

Light‐harvesting is of vital importance for many events, such as photosynthesis. To efficiently gather and transfer solar energy, delicate antenna is needed, which has been achieved by algae and plants. However, construction of efficient light‐harvesting systems using multiple, artificial building blocks is still challenging. Here, blue‐emitting organosilicone capsules containing carbon dots (denoted as CDs‐Si) in ethanol are prepared, which can effectively transfer energy to green‐emitting (silicone‐functionalized bodipy, Si‐BODIPY) or red‐emitting (rhodamine b, RhB) dyes. In ternary system, sequential Förster resonance energy transfer from CDs‐Si to Si‐BODIPY and further to RhB is realized, which is accompanied with a less pronounced, parallel FRET directly from CDs‐Si to RhB. The overall efficiency of energy transfer reaches ≈86%. By introducing a photoswitch (1,2‐bis(2,4‐dimethyl‐5‐phenyl‐3‐thienyl)‐3,3,4,4,5,5‐hexafluoro‐1‐cyclopentene, DAE) to the system, the emission becomes switchable under alternative illumination with UV and visible light, leading to the formation of smart artificial light‐harvesting systems.

## Graphical abstract

A new generation of donors is developed by adopting the advantages of CDs and organosilicone that exhibits *n*‐PL. The rather broad emission of the donor enables the construction of ALHSs with both green‐ and red‐emitting dyes. Further introduction of a photochromic molecules led to the formation of photoswitchable ALHSs.

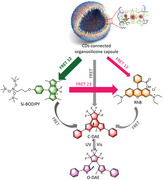

## 1 Introduction

Photosynthesis, a biological process of converting light energy into chemical energy, is the largest scale of chemical reaction naturally occurring in the biosphere that produces necessary nutrients for animals and human beings.^[^ ^1^ ^]^ As sunlight has a rather broad distribution of the wavelength and its energy density is in many cases not enough, photosynthetic organisms have developed antenna to harvest light, after which sequential Förster resonance energy transfer (FRET)^[^ ^2^ ^]^ occurs toward the photosynthetic center. Inspired by natural LHSs, many artificial LHSs (ALHSs) exhibiting sequential FRET have been developed.^[^ ^3^, ^4^, ^5^, ^6^, ^7^, ^8^, ^9^, ^10^, ^11^, ^12^, ^13^, ^14^, ^15^, ^16^ ^]^ These ALHSs typically use antenna with explicit molecular structures, including derivatives of planar chromophores,^[^ ^3^, ^4^ ^]^ metallacycles,^[^ ^5^, ^6^, ^7^, ^8^, ^9^, ^10^ ^]^ and propeller‐type molecules^[^ ^11^, ^12^, ^13^, ^14^, ^15^ ^]^ with aggregation‐induced emission (AIE).^[^ ^16^, ^17^, ^18^ ^]^ In comparison, the antenna in natural LHSs have much more complicated structures. For example, in algae and plants, the antenna normally comprises hundreds of pigments, which distribute on rigid protein scaffolds in a well‐organized manner through noncovalent interactions.^[^ ^19^, ^20^ ^]^ Inspired by this, antenna containing multiple components, such as photoluminescent (PL) nanomaterials, could also be popular, which has however been rarely investigated.^[^ ^21^ ^]^

During the past decades, we have witnessed the encouraging discoveries of new photoluminescent (PL) materials. As a typical example, carbon dots (CDs) are a class of PL carbon nanomaterials and easy to prepare.^[^ ^22^, ^23^, ^24^, ^25^ ^]^ They comprise building blocks with different sizes and surface states, leading to a rather broad emission which is advantageous for light harvesting. Another example is nonconventional photoluminescence (*n*‐PL) from clustering of molecules free of conventional chromophores but rich in electron‐abundant heteroatoms such as N, Si, and O, which has been noticed for a long history but reattracted attention from the scientific community only recently.^[^ ^26^ ^]^ CDs and materials exhibiting *n*‐PL provide new opportunities for the construction of ALHSs, but, big challenges also remain. As is well‐known, a good donor in ALHSs needs not only a satisfactory fluorescence quantum yield (φ), but also the capability of undergoing supramolecular interaction with the acceptor. Although a large number of CDs have been reported, candidates meeting these requirements are quite rare.^[^ ^27^, ^28^ ^]^ For materials exhibiting *n*‐PL, typically the emission is located in blue region with relatively low φ, which is also hard to be utilized in the construction of LHSs. To the best of our knowledge, there is no report clearly demonstrating the role played by materials exhibiting *n*‐PL in FRET.

**Scheme 1:**
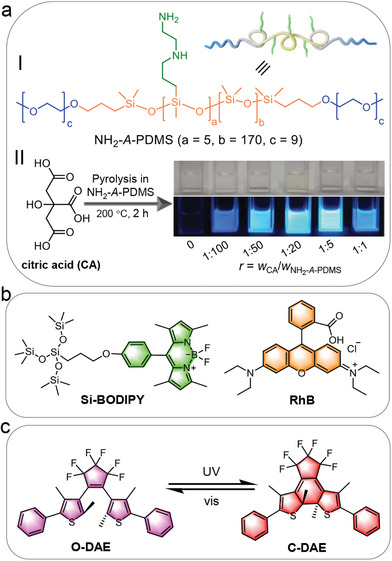
Structures of the building blocks adopted in current study. a) Engineering of the donor. I: structure of the amine‐modified amphiphilic polydimethylsiloxane (NH_2_‐A‐PDMS). II: Synthesis of carbon dots from citric acid in NH_2_‐A‐PDMS. On the right are photos of the product in EtOH (2 mg mL^−1^), taken under roomlight (top) and 365 nm UV irradiation (bottom). b) Structures of the silicone‐modified BODIPY (Si‐BODIPY, the relay acceptor) and RhB (the final acceptor). c) Structural change of the photoswitch DAE.

Clearly, to successfully utilize CDs and *n*‐PL materials in ALHSs, smart design is needed to evoke their advantages while at the same time, suppress their shortcomings. Keeping this in mind, in this work we first synthesized an amine‐modified, amphiphilic polydimethylsilixane (NH_2_‐A‐PDMS, I in **Scheme** **1**a), which showed concentration‐dependent aggregation behavior in ethanol (EtOH) and could form capsules. Through an in situ pyrolysis, CDs derived from citric acid (CA) were anchored onto the chains of NH_2_‐A‐PDMS. In the complex (denoted as CDs‐Si hereafter), NH_2_‐A‐PDMS acted as a skeleton to suppress the aggregation‐caused quenching of CDs, while CDs served as nodes to enhance the *n*‐PL of NH_2_‐A‐PDMS. These advantages impart CDs‐Si bright blue emission (II in Scheme 1a). The capsules formed by CDs‐Si were used as a donor, which was coupled with green‐emitting (Si‐BODIPY, Scheme 1b) and red‐emitting (RhB, Scheme 1b) dyes. The broad emission of the capsule covers the absorption of both dyes, which facilitated FRET in CDs‐Si/Si‐BODIPY and CDs‐Si/RhB binary systems. In CDs‐Si/Si‐BODIPY/RhB ternary system, a sequential FRET from CDs‐Si to Si‐BODIPY and further to RhB was realized together with a parallel FRET directly from CDs‐Si to RhB, giving an overall energy transfer efficiency up to ≈86%. By introducing a photoswitch (1,2‐bis(2,4‐dimethyl‐5‐phenyl‐3‐thienyl)−3,3,4,4,5,5‐hexafluoro‐1‐cyclopentene, DAE, Scheme 1c) to the system, the emission became switchable under alternative illumination with UV and visible light, leading to the formation of smart ALHSs.

## 2 Result and Discussion

### 2.1 Preparation of the Donor

**Figure 1:**
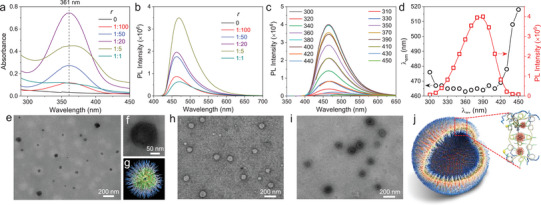
Optical properties and self‐assembly behavior of the donor. a) UV‐vis absorption of CDs‐Si (1 mg mL^−1^ in EtOH) obtained at selected *r*. b) Emission of CDs‐Si (2 mg mL^−1^ in EtOH, λ_ex_ = 390 nm) obtained at selected *r*. c) Emission of CDs‐Si (2 mg mL^−1^ in EtOH, *r* = 1:5) at varying λ_ex_. d) Statistics of the λ_em_ and PL intensity given in graph c. e) A typical TEM image of the dilute EtOH solution of NH_2_‐A‐PDMS in EtOH (0.02 mg mL^−1^), showing the formation of micelles. f) Magnified image of a single micelle, note the slightly light color in the center was caused by the irradiation of the electron beam. g) A model of the micelle. h) TEM image of NH_2_‐A‐PDMS in EtOH (0.2 mg mL^−1^). i) TEM image of CDs‐Si in EtOH (0.2 mg mL^−1^, *r* = 1:5). j) Proposed structure of the capsule formed by CDs‐Si.

The synthetic procedures of NH_2_‐A‐PDMS are given in Scheme S1 (Supporting Information). It has a linear structure with two terminal, short hydrophilic polyoxyethylene chains. In the middle is a long PDMS chain, on which five short, amine‐terminated side chains were randomly inserted. Although NH_2_‐A‐PDMS contains Si, N, and O, it exhibits negligible emission both in solvent‐free state and dilute solution (in EtOH), indicating that the clustering of these heteroatoms is not enough for effective through space conjugation (TSC).^[^ ^29^ ^]^ Hybrid material of CDs‐Si was prepared by a solvent‐free pyrolysis of CA in NH_2_‐A‐PDMS. From Fourier transform infrared spectra (FTIR, Figure S1, Supporting Information), the content of the carbonyl group increases continuously with increasing weight ratio of CA to NH_2_‐A‐PDMS (*r*). From UV–vis measurements, NH_2_‐A‐PDMS only shows absorption in the UV region (Figure S2, Supporting Information), while for CDs‐Si a new absorption peak centered at 361 nm was detected (**Figure** **1**a). Meanwhile, a broad emission was detected starting from ≈425 nm and tailing to ≈600 nm. During pyrolysis, condensation among the active groups of CA and NH_2_‐A‐PDMS will occur, which will close the gap among the electron‐abundant heteroatoms. As the pyrolysis temperature adopted in current study (200 °C) is well above that of the decomposition and carbonization of CA (≈180 °C), formation of CDs is expected, although direct proofs from imaging study is hard to obtain as the CDs are small and embedded in the PDMS network. Thus, CDs‐Si represents a complicated donor, with PL contributions from both CDs and *n*‐PL.

Steady‐state fluorescence measurements showed that the emission of CDs‐Si depends on several parameters, including *r* adopted in the pyrolysis, the concentration of the product (*c*_CDs‐Si_) and the wavelength of excitation (λ_ex_), with the strongest emission occurring at *r* = 1:5, *c*_CDs‐Si_ = 2 mg mL^−1^ and λ_ex_ = 390 nm (Figure 1b–d; Figure S3, Supporting Information). The φ of CDs‐Si was determined to be 47.5%. The formation of the amide groups leads to a more condensed crosslinking of the polymer chains, which, combined with the increase of the number density of the oxygen atoms in the system, is expected to enhance the *n*‐PL of the system.^[^ ^30^, ^31^ ^]^ As CDs are well dispersed and their aggregation was largely avoided, the aggregation‐caused quenching (ACQ) effect^[^ ^32^, ^33^ ^]^ could be greatly suppressed. These two effects account for the bright blue emission of the CDs‐connected organosilicone network.

Bearing an amphiphilic structure, NH_2_‐A‐PDMS is surface active in EtOH. At a concentration of 2 mg mL^−1^, the surface tension of the EtOH solution reaches 20.7 mN m^−1^ (Figure S4, Supporting Information), which is lower than that of pure EtOH (21.4 mN m^−1^). Imaging studies by transmission electron microscopy (TEM) observations revealed that it undergoes concentration‐dependent aggregation behavior in EtOH. In dilute solution (0.02 mg mL^−1^), formation of micelles was confirmed (Figure 1e–g). The five amine‐terminated side chains probably bring some solvent molecules inside the micelles, accounting for their sensitivity to the electron beam. At a concentration of 0.2 mg mL^−1^ and 0.5 mg mL^−1^, capsules were clearly observed (Figure 1h; Figure S5, Supporting Information) with an averaged diameters of ≈75 nm. After CDs were anchored, the CDs‐Si preserved the capability to form capsules. A typical TEM image obtained at *c*_CDs‐Si_ = 0.2 mg mL^−1^ is given in Figure 1i and an illustration of the proposed structure of the capsule is shown in Figure 1j. Compared to the capsules formed by NH_2_‐A‐PDMS with the same concentration, the capsules formed by CDs‐Si are larger (Figure S6, Supporting Information). Dynamic light scattering (DLS) measurement gave an averaged hydrodynamic radius of ≈200 nm (Figure S7, Supporting Information), which is slightly larger than that obtained from TEM observations, presumably due to the solvation of the capsules in EtOH. Capsules were also detected in other concentrations, such as 0.02 mg mL^−1^ and 2.0 mg mL^−1^ (Figure S8, Supporting Information). No obvious variation of the sizes could be noticed, indicating that the crosslinking of NH_2_‐A‐PDMS by CDs makes the capsules robust. It should be noted that at 2 mg mL^−1^ (the concentration we selected for the construction of ALHSs), the number density of the capsules is even smaller, as CDs‐Si easily forms film upon evaporation of EtOH during sample preparation for both TEM and scanning electron microscopy (SEM) observations. Interestingly, even within the film, the survived capsules could also be found (Figure S9, Supporting Information).

### 2.2 Construction of ALHSs with Sequential and Parallel FRET

**Figure 2:**
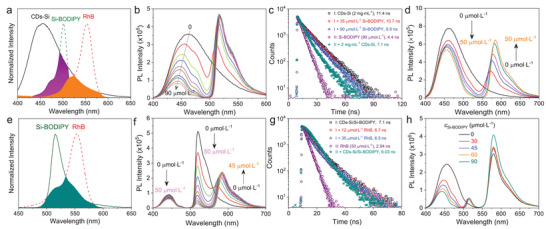
Evidences of the FRET Processes involved among the donor and the two acceptors. Samples were excited at 390 nm unless otherwise stated. a) Normalized emission of CDs‐Si and the absorption of Si‐BODIPY and RhB. The overlapped areas are highlighted. b) Variation of the emission of CDs‐Si (2 mg mL^−1^) upon the addition of Si‐BODIPY (with a step of 10 µmol L^−1^). c) PL decay recorded at λ_em_ = 460 nm for CDs‐Si (2 mg mL^−1^) without and with 35 µmol L^−1^ or 90 µmol L^−1^ Si‐BODIPY. Also shown are results recorded at λ_em_ = 520 nm from Si‐Bodipy (90 µmol L^−1^) without and with 2 mg mL^−1^ CDs‐Si. d) Variation of the emission of CDs‐Si (2 mg mL^−1^) upon the addition of RhB (with a step of 10 µmol L^−1^). e) Normalized emission of Si‐Bodipy (λ_ex_ = 460 nm) and the absorption of RhB. The overlapped area is highlighted. f) Variation of the emission of the sample containing 2 mg mL^−1^ CDs‐Si and 90 µmol L^−1^ Si‐BODIPY upon the addition of RhB (with a step of 10 µmol L^−1^). g) PL decay recorded at λ_em_ = 520 nm for the mixture of CDs‐Si (2 mg mL^−1^)/Si‐Bodipy (90 µmol L^−1^) without and with 35 µmol L^−1^ RhB, Also shown are results recorded at λ_em_ = 580 nm from RhB (50 µmol L^−1^) without and with the mixture of CDs‐Si (2 mg mL^−1^)/Si‐Bodipy (90 µmol L^−1^). h) Variation of the emission of CDs‐Si (2 mg mL^−1^) and RhB (50 µmol L^−1^) upon the addition of Si‐BODIPY.

**Figure 3:**
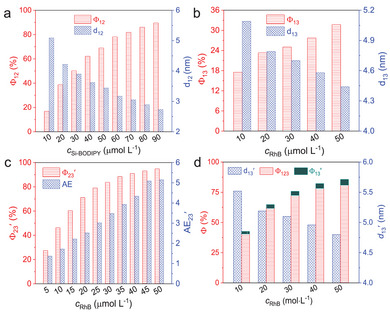
Plot of selected parameters involved in the FRET Processes. Details of the samples have been given in a) Figure 2b, b) Figure 2c, c,d) Figure 2f, respectively.

To construct ALHSs with sequential FRET, two acceptors were selected. The relay acceptor is a green‐emitting BODIPY derivative functionalized with a branched silicone chain (Si‐BODIPY, Scheme 1b). The detailed synthetic procedures of Si‐BODIPY were given in Scheme S2 (Supporting Information), and its structure was unambiguously confirmed by ^1^H NMR, ^13^C NMR and HR‐ESI‐MS. In EtOH, it emits green light with a φ of 40.57% and an averaged lifetime (<τ>) of 4.4 ns (Figures S10–S12 and Table S1, Supporting Information). While the position of the emission basically does not change with λ_ex_, the PL intensity varies obviously with a maximum at λ_ex_ = 460 nm and a secondary one at λ_ex_ = 390 nm (Figure S10, Supporting Information). The final acceptor is rhodamine B (RhB, Scheme 1b), a well‐known dye with red‐emission in EtOH (Figures S13 and S14, Supporting Information). FRET among CDs‐Si and the two acceptors were investigated by steady‐state and time‐resolved fluorescence measurements, which are summarized in **Figure** **2**. Further analysis of the data with Förster's Theory yielded important parameters, including rate constant of the energy transfer (*k*_ET_), energy transfer efficiency (Φ), distance (*d*) of the donor/acceptor pair, and antenna effect (AE), which are given in Figure 3 and Tables S2–S8 (Supporting Information). Titration of a 2 mg mL^−1^ solution of CDs‐Si in EtOH with Si‐BODIPY (Figure 2b) induces a gradual increase of the emission in the green light region with a slight bathochromic shift (Figure S15, Supporting Information) when excited at 390 nm, while the emission of CDs‐Si continuously decreases (Figure 2b; Figure S16, Supporting Information). A hypochromatic shift (up to 21 nm) was noticed for CDs‐Si together with the shrinking of the band width at half height from 80 nm to 36.5 nm, as the overlapping of its emission with the absorption of Si‐BODIPY mainly occurs in the long wavelength range, seen from Figure 2a and Figure S17 (Supporting Information). Calculations gives a Fröster radius (R_0_) of 3.9 nm for CDs‐Si/Si‐BODIPY pair. At *c*_Si‐BODIPY_ = 10 µmol L^−1^, the averaged distance between CDs‐Si and Si‐BODIPY (*d*_12_) was calculated to be 5.09 nm (**Figure** **3**a; Table S2, Supporting Information), which falls within the range of efficient FRET (0.5–1.5R_0_).^[^ ^34^, ^35^, ^36^, ^37^ ^]^ With increasing *c*_Si‐BODIPY_, *d*_12_ continuously decreases and reaches a value of 2.73 nm at *c*_Si‐BODIPY_ = 90 µmol L^−1^, which leads to improvements of Φ (Φ_12_) from 16.8% to 89.5%, *k*_ET_ from 1.8 × 10^7^ s^−1^ to 7.5 × 10^8^ s^−1^, and AE (AE_12_) from 0.10 to 0.55 (Figure 3a; Figure S18 and Tables S2 and S3, Supporting Information). The FRET changes the <τ> of both the donor and the acceptor. As shown in Figure 2c, <τ> of 2 mg mL^−1^ CDs‐Si decreases from 11.4 ns to 9.9 ns after the addition of 90 µmol L^−1^ Si‐BODIPY, while that of 90 µmol L^−1^ Si‐BODIPY increases from 4.4 to 7.1 ns after the addition of 2 mg mL^−1^ CDs‐Si.

The rather broad emission of CDs‐Si also leads to a considerable overlapping area with the absorption of RhB (Figure 2a; Figure S19, Supporting Information). Calculations give an even larger *J*(λ) (6.65×10^14^ M^−1^ cm^−1^ nm^−4^) than that of the CDs‐Si/Si‐BODIPY pair (4.13×10^14^ M^−1^ cm^−1^ nm^−4^). R_0_ of the CDs‐Si/RhB pair is also larger, which is 4.2 nm. The FRET from CDs‐Si to RhB was confirmed by titration (Figure 2d) and time‐resolved fluorescence (Figure S20, Supporting Information) measurements. Compared to the CDs‐Si/Si‐BODIPY system, key parameters including k_ET_, Φ_13_ and AE_13_ are, however, lower (Figure 3b; Figure S21, Tables S4 and S5, Supporting Information) due to the larger distance between CDs‐Si and RhB (*d*_13_, Figure 3b; Table S4, Supporting Information).

Considering that the emission of Si‐BODIPY overlaps well with the absorption of RhB (Figure 2e), a sequential FRET was expected in the ternary system of CDs‐Si/Si‐BODIPY/RhB. Calculations gives the largest R_0_ (5.1 nm) among the three donor/acceptor pairs, which means that occurrence of FRET is possible within a large range of distance (2.58 ≈ 7.74 nm). Titration with RhB was carried out on two series of samples with 2 mg mL^−1^ CDs‐Si and 90 µmol L^−1^ or 50 µmol L^−1^ Si‐BODIPY. In both cases, emission in the red light region increases in expense of the emission in the green light region (Figure 2f; Figure S22, Supporting Information), and <τ> of the CDs‐Si/Si‐BODIPY mixture decreases with increasing *c*_RhB_ while that of RhB increases after the addition of the CDs‐Si/Si‐BODIPY mixture (Figure 2g). Calculations gave high efficiencies of this FRET (denoted as Φ_23_′), with the maximums to be 94.8% for samples with 90 µmol L^−1^ Si‐BODIPY (Figure 3c; Table S6, Supporting Information), and 93.8% for those with 50 µmol L^−1^ Si‐BODIPY (Figure S23 and Table S7, Supporting Information), respectively. Other advantages include the improved k_ET_ and AE (AE_23_′) (Figure 3c; Figures S23 and S24, Tables S6 and S7, Supporting Information). Experiments were also carried out by titrating a mixture of CDs‐Si/RhB with Si‐BODIPY. As shown in Figure 2h, the addition of Si‐BODIPY does not lead to an increase of its own emission. Instead, it enhances the emission of RhB in expense of that of CDs‐Si, indicating the important role played by the relay acceptor to amplify the light harvesting.

From Figure 2f and Figure S22 (Supporting Information), one can see that the blue emission from CDs‐Si also decreases with the addition of RhB, albeit with a smaller content compared to the decrease of the green emission. This means that a parallel FRET directly from CDs‐Si to RhB also occurs in ternary systems, which will compete with the FRET from CDs‐Si to Si‐BODIPY. Parameters of these two FRET processes were amended for the ternary system with 90 µmol L^−1^ (Table S8, Supporting Information). The Φ of the sequential FRET (denoted as Φ_123_) from CDs‐Si to Si‐BODIPY and further to RhB was calculated by Φ_23_′ × Φ_12_′, and the overall Φ (Φ_overall_) was obtained by Φ_123_ + Φ_13_′ (Figure 3d). One can see that the sequential FRET is dominant, which is consistent with systems comprising two acceptors where FRET from the donor to the final acceptor is usually less pronounced.^[^ ^38^ ^]^With increasing *c*_RhB_, the distance between CDs‐Si and Si‐BODIPY (*d*_12_′) remains almost unchanged, while that between CDs‐Si and RhB (*d*_13_′) decreases obviously (Figure 3d; Table S8, Supporting Information), indicating that that arrangement of the donor and the acceptors becomes more compact. TEM observations on CDs‐Si/Si‐BODIPY binary mixture revealed the capsules have sizes smaller than those before the addition of Si‐BODIPY, which are swollen upon dilution (Figure S25, Supporting Information). This variability upon the change of solution parameters is common for supramolecular structures formed by multiple components. Capsules were also detected in CDs‐Si/Si‐BODIPY/RhB ternary system (Figure S26, Supporting Information). From SEM mapping on the films prepared by drying the EtOH solution of the binary and ternary mixtures, boron and fluorine from Si‐BODIPY, and chlorine from RhB distribute evenly with no macroscopic phase separation (Figures S27 and S28, Supporting Information). These results indicate that the acceptors have integrated into the capsules formed by CDs‐Si driven by noncovalent interactions including solvophobic effect, π‐π stacking as well as electrostatic interaction proved by the change of zeta potential measurements (Figure S29, Supporting Information). It should be emphasized that the amphiphilicity of CDs‐Si in EtOH, with the strong evidence of capsule formation, is one of the key factors for the successful FRET. In control experiment where CDs‐Si was replaces by another type of blue‐emitting CDs, no FRET process could be realized (Figure S30, Supporting Information).

**Figure 4:**
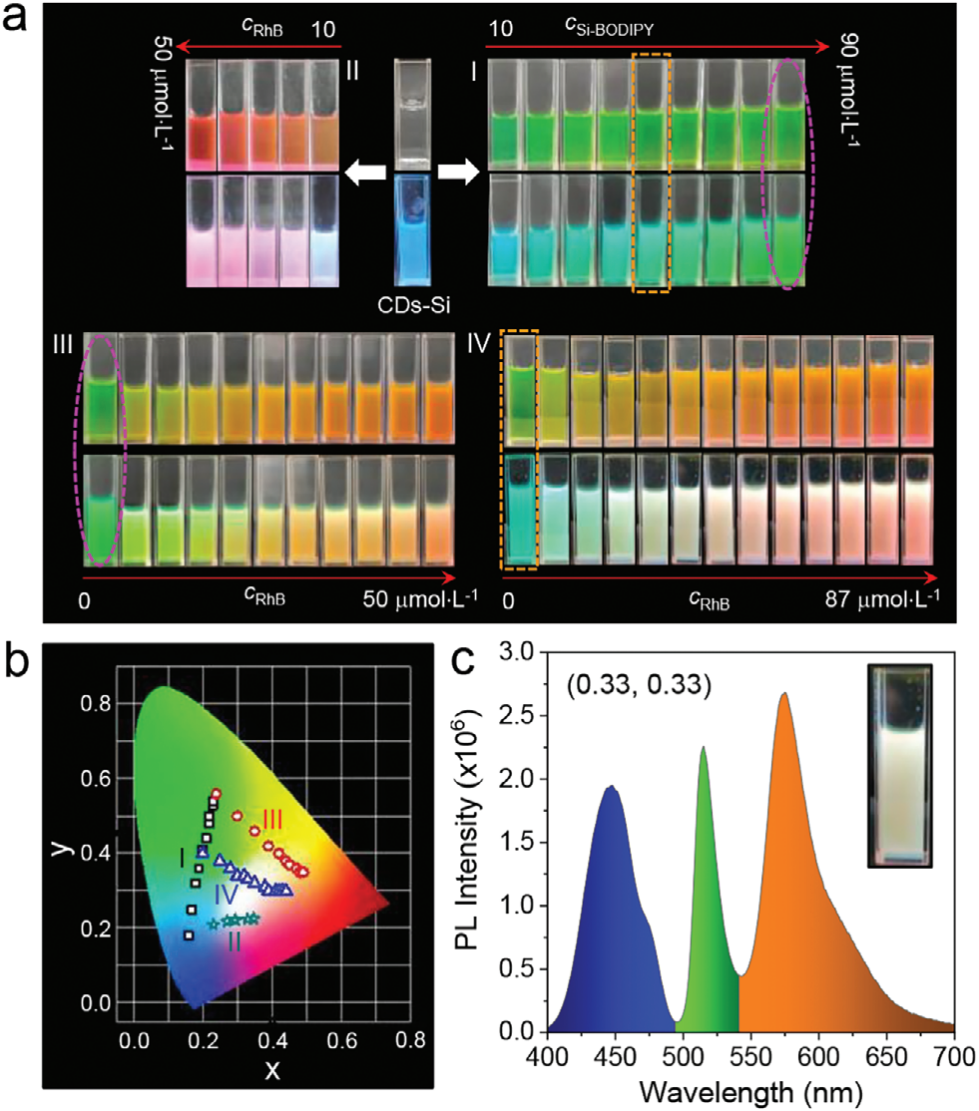
Color change induced by FRET. a) Photos of the four series of samples under room light (top rows) and 365 nm UV (bottom rows). Details of the samples can be found in Figure 2b (series I), Figure 2d (series II), Figure 2f (series III) and Figure S22 (Supporting Information) (series IV). b) The CIE coordinates of the four series of samples. c) The emission of the white‐light emission sample, with a composition of 2 mg mL^−1^ CDs‐Si, 50 µmol L^−1^ Si‐BODIPY and 23.8 µmol L^−1^ RhB. Insets are the CIE coordinates and photo of the sample under 365 nm UV.

The FRET among CDs‐Si, Si‐BODIPY, and RhB leads to interesting color change both under room light and 365 nm UV irradiation (**Figure** **4**a). From the variation of the CIE coordinates (Figure 4b), one can see that the samples cover a large area, including regions of the blue, green, yellow, and red light. Specifically, white‐light emission with a perfect CIE coordinate of (0.33, 0.33) was obtained (Figure 4c), providing great potential applications in organic light‐emitting diodes (OLEDs).

### 2.3 Construction of ALHSs with Tunable FRET

**Figure 5:**
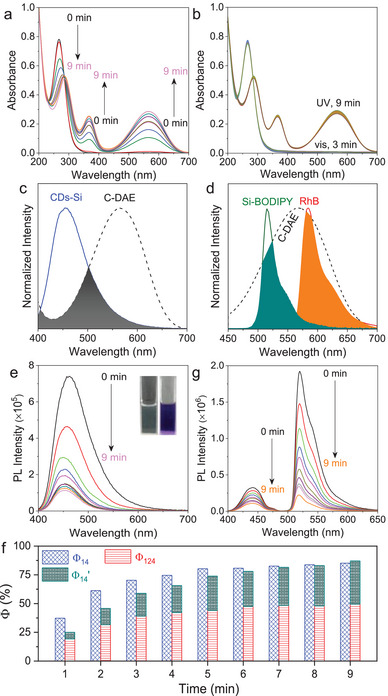
Control of the emission by the photoswitch DAE in binary and ternary mixtures. a) Variation of the absorption of DAE (100 µmol L^−1^ in EtOH) under continuous irradiation of 365 UV light. b) UV‐vis absorption of DAE upon alternative irradiation of UV and visible light (up to ten cycles). c,d) Overlapping of the absorption of C‐DAE with the emission of CDs‐Si (λ_ex_ = 390 nm), Si‐BODIPY (λ_ex_ = 460 nm) and RhB (λ_ex_ = 490 nm). e) Variation of the emission under continuous irradiation of 254 UV light of a binary mixture containing 2 mg mL^−1^ CDs‐Si and 200 µmol L^−1^ DAE (λ_ex_ = 390 nm). Insets are photos of the sample recorded at 0 (left) and 9 (right) min under room light. f) Statistics of Φ for the FRET from CDs‐Si to Si‐BODIPY in the binary (Φ_14_) and ternary (Φ_14_′) mixture, and Φ of the sequential FRET (Φ_124_) in the ternary mixture. g) Variation of the emission under continuous irradiation of 254 UV light of a ternary mixture containing 2 mg mL^−1^ CDs‐Si, 90 µmol L^−1^ Si‐BODIPY and 200 µmol L^−1^ DAE (λ_ex_ = 390 nm).

To engineer smart ALHSs responsive to external stimuli, a photochromic molecule (DAE) was introduced, which undergoes a reversible cyclization reaction under alternative UV and visible (vis) light irradiation (Scheme 1c).^[^ ^39^, ^40^, ^41^, ^42^ ^]^ The change in structure leads to strong variations in its absorption. As shown in **Figure** **5**a, when being irradiated with 254 nm UV light for 9 min, the absorption in the 300–400 nm and 500–600 nm range continuously increases, while that in the 250–300 nm range gradually decreases, which caused by a structural transformation from the opened form (O‐DAE) to the closed one (C‐DAE). The opposite transition was achieved under illumination with an incandescent lamp (10 W) for 3 min. This process maintains high stability, up to at least the ten cycles without fatigue (Figure 5b; Figure S31, Supporting Information).

While O‐DAE is optically transparent at wavelengths above 350 nm, C‐DAE has rich absorption in the visible region which exhibits considerably large overlapping areas with the donor (CDs‐Si, Figure 5c) and the two acceptors (Si‐BODIPY and RhB, Figure 5d). When O‐DAE (200 µmol L^−1^) was added to an EtOH solution of CDs‐Si (2 mg mL^−1^), effective fluorescence quenching was observed under continuous irradiation with a 254 nm UV light (Figure 5e) together with a significantly aggravated color of the sample (inset of Figure 5e). Time‐resolved fluorescence measurement showed that <τ> decreased from 11.4 ns at 0 min to 10.8 ns at 9 min (Figure S32, Supporting Information), indicating that the energy transfer from CDs‐Si to C‐DAE belongs to FRET. The Φ (denoted as Φ_14_) is up to 85.2% within the first 9 min (Figure 5f). In ternary system with additional presence of Si‐BODIPY (90 µmol L^−1^), fluorescence quenching of both CDs‐Si and Si‐BODIPY was observed (Figure 5g). Analysis on the time‐dependent PL intensity showed that the quenching of the fluorescence of Si‐BODIPY is faster than that of CDs‐Si (Figure S33, Supporting Information), and PL decay recorded at λ_em_ = 520 nm revealed that the initial <τ> (7.2 ns) decreased to 6.5 ns at 4 min and further to 6.3 ns at 9 min (Figure S34, Supporting Information). These results unambiguously confirmed that in this ternary system, besides FRET from CDs‐Si to C‐DAE, an additional FRET from Si‐BODIPY to C‐DAE also occurred. The Φ of the sequential FRET from CDs‐Si to Si‐BODIPY and then to C‐DAE (Φ_124_ = Φ_24_′ × Φ_12_′′), and that from CDs‐Si directly to C‐DAE (denoted at Φ_14_′) were calculated and given in Figure 5f. The highest Φ_overall_ (= Φ_124_ + Φ_14_′) reaches 87.1% at 9 min. Comparison between Figure 3d and Figure 5f reveals that the CDs‐Si/Si‐BODIPY/C‐DAE mixture has a lower proportion of the sequential FRET than that of CDs‐Si/Si‐BODIPY/RhB system, which means that we can tune the channel of the FRET by changing the components.

**Figure 6:**
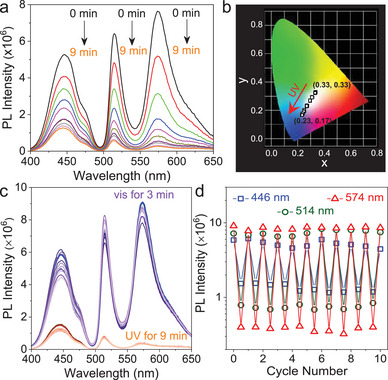
Control of the emission (λ_ex_ = 390 nm) by the photoswitch DAE in quaternary mixture. a,b) Variations of the emission and corresponding CIE coordinate of the sample containing 2 mg mL^−1^ CDs‐Si, 50 µmol L^−1^ Si‐BODIPY, 23.8 µmol L^−1^ RhB and 200 µmol L^−1^ DAE under continuous irradiation of 254 UV light. c,d) Emission curves and statistics of the intensity of the three peaks of the sample upon alternative irradiation of UV and visible light (up to ten cycles). The sample has the same composition with that in a.

**Scheme 2:**
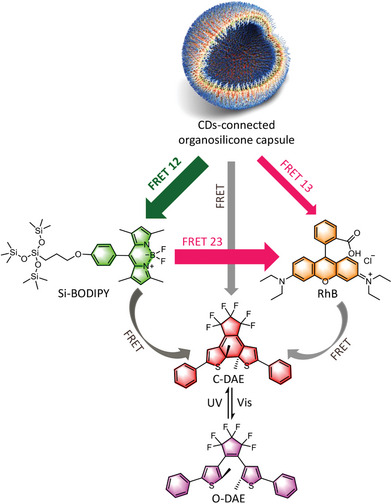
Illustration of the energy transfer occurred in current system.

Now, we discuss the FRET processes occurring in the quaternary system of CDs‐Si/Si‐BODIPY/RhB/DAE. Investigations were performed on two samples. The first one contains 50 µmol L^−1^ Si‐BODIPY and 23.8 µmol L^−1^ RhB which gives white light emission. As seen from **Figure** **6**a, emission from the three chromophores all decreased upon the irradiation of 254 nm UV. Analysis on the time‐dependent PL intensity showed that the quenching of the fluorescence follows the order of RhB > Si‐BODIPY > CDs‐Si (Figure S35, Supporting Information), indicating that FRET from RhB to C‐DAE also occurred. Results from another sample containing 90 µmol L^−1^ Si‐BODIPY and 50 µmol L^−1^ RhB gave the same conclusion (Figure S36, Supporting Information). Thus, in the quaternary system, a sequential FRET from CDs‐Si to Si‐BODIPY to RhB and finally to C‐DAE was constructed. Meanwhile, FRET directly from CDs‐Si and Si‐BODIPY to C‐DAE could also happen parallelly. This multilevel FRET to C‐DAE guarantees a high efficiency of fluorescence quenching, and the excessive consumption of the emission from longer wavelengths causes a color change from a perfect white light (0.33, 0.33) to a blue light (0.23, 0.17) during quenching (Figure 6b). The FRET involved DAE, together with the pristine FRET among CDs‐Si, Si‐BODIPY, and RhB (**Scheme** **2**), makes the quaternary system quite complicated where explicitly quantifying the contribution of each FRET becomes difficult. Originated from the reversible response of DAE to external light irradiation, the DAE‐containing ALHSs exhibited good reversibility, which could be manipulated for at least ten times without fatigue (Figure 6c,d; Figure S37, Supporting Information).

## 3 Conclusion

In summary, we have developed a new generation of donors by adopting the advantages of CDs and organosilicone that exhibits *n*‐PL. The rather broad emission of the donor enables us to construct ALHSs with both green‐ and red‐emitting dyes. In ternary systems, sequential and parallelly‐occurring FRET process was realized, which is beneficial to gather and transfer light energy with a high efficiency. By adjustment of the composition, samples emitting different colors were obtained under 365 nm UV, including the perfect white light. Further introduction of a photochromic molecules led to the formation of photoswitchable ALHSs. This work indicates that the newly‐discovered PL materials, such as CDs and molecules exhibiting *n*‐PL, could be good candidates for the construction of ALHSs. In future, ALHSs in aqueous environment or solvent‐free state should be designed to fully excavate the potential of these PL nanomaterials toward mimicking natural LHSs.

## Conflict of Interest

The authors declare no conflict of interest.

## Author Contributions

L.Y. performed investigation, data curation, and writing – original draft. H.L. performed resources and formal analysis. N.F. performed CDs‐Si preparation, resources, and formal analysis. G.Y. performed investigation. X.X. performed project administration and supervision. J.H. performed supervision. H.‐G.L. performed supervision, conceptualization, and writing – review & editing.

## Supporting information

Supporting Information

## Data Availability

The data that support the findings of this study are available from the corresponding author upon reasonable request.
