# Supplementary material for: Multi‐Step and Switchable Energy Transfer in Photoluminescent Organosilicone Capsules
Source: Adv Sci (Weinh). 2024 Jun 18;11(31):2402565. doi: 10.1002/advs.202402565 (PMC11336914; doi:10.1002/advs.202402565)
Supplement: Supplementary file 1 — Supporting Information [file ADVS-11-2402565-s001.docx]

**Multi-Step and Switchable Energy Transfer in Photoluminescent** **Organosilicone Capsules**

Longyue Yu,^a^ Hailong Liu,^b^ Ning Feng,^a^ Gang Yi,^b^ Xia Xin,^c^ Jingcheng Hao,^a^ Hongguang Li^a,^^[[1]](#footnote-1)^*

*^a^ Key Laboratory of Colloid and Interface Chemistry, Ministry of Education, School of Chemistry and Chemical Engineering, Shandong University, Jinan, Shandong, 250100, China*

*^b.^Shandong Key Laboratory of Advanced Organosilicon Materials and Technologies,* Zibo, 256401, China

*^c^* *National Engineering Research Center for Colloidal Materials, Shandong University, Jinan, 250100, China*

**1. Experimental section**

**1.1 Materials and general methods**

All chemicals were commercially acquired. 1,2-bis(2,4-dimethyl-5-phenyl-3-thienyl)-3,3,4,4,5,5-hexafluoro-1-cyclopentene (DAE), trifluoroacetic acid (TFA), dichloro-5,6-dicyanobenzoquinone (DDQ), Triethylamine (TEA), boron trifluoride ether (BF_3_⋅OEt_2_) were obtained from Macklin with AR grade; potassium carbonate (K_2_CO_3_), potassium iodide (KI), sodium chloride (NaCl), anhydrous sodium sulfate (Na_2_SO_4_), sodium bicarbonate (NaHCO_3_), and Organic solvents including N, N-dimethylformamide (DMF), dichloromethane (CH_2_Cl_2_), petroleum ether were obtained from local supplier with AR grade.

^1^H NMR and ^13^C NMR were performed using a Bruker 400 MHz instrument. The corresponding chemical shifts were calculated with regard to the residual solvent signals. Coupling constants (J) and chemical shifts (d) were reported in Hz and ppm, respectively. Symbols “s”, “d”, “m”, and “br” were used to indicate single, double, and multiple multiplicities and broad signals, respectively. Mass spectrum were obtained on an Agilent 6510Q-TOF. UV-vis spectra were recorded in a quartz cell (light path 1 mm) on a U4100 UV-vis spectrometer (Hitachi, Japan). Steady-state fluorescence spectra were acquired on a FLS920 (Edinburg Instruments Ltd., Livingstone, UK) loaded on quartz plates and a microplate spectrometer (TECAN, SparkControl Magellan), loaded on Costar black plates. Quantum yields were also measured on the FLS920, which was loaded on a quartz plate and polystyrene plate, respectively. The fluorescence spectra were recorded using LS-55 spectrofluorometer (PerkinElmer, Waltham, MA, USA) and an Edinburgh Instruments FLS920 luminescence spectrometer (xenon lamp, 450 W). Fourier transform infrared (FT-IR) spectra were recorded on a Tensor II spectrophotometer (Bruker, Germany) while samples were carried with KBr powder. In field-emission scanning electron microscopy (SEM) observations, two drops of suspension were placed on a silica wafer. Then, the silica wafer was dried under infrared lamp for 35 minutes. The samples were observed by Hitachi SU8010 with an accelerating voltage at 10 kV. The zeta potential and DLS measruemnts were carried out using the NanoZS (Malvern, UK) instrument with designated cells, all samples were filtered through a 2.2 μm PTFE membrane before measurements.

Femtosecond TA spectroscopy measurements were performed on an Ultrafast Helios pumpprobe system in collaboration with a regenerative amplified laser system. An 800 nm pulse with 100 fs pulse width, an energy of 7 mJ per pluse, and a repetition rate of 1 kHz was generated by a Ti:sapphire amplifier (Astrella, Coherent) and split into two parts. One of the beams was coupled into an optical parametric amplifier (TOPAS, Coherent) to generate the pump pulses at 800 nm and the other part was focused onto a sapphire plate and a YAG plate to generate a broad band of 400–1200 nm probe light. The time delay between pump and probe was controlled by a motorized optical delay line with a maximum delay time of 8 ns. Samples were fabricated on quartz substrates and were encapsulated in a nitrogen-filled glovebox with epoxy resin to minimize air-induced degradation.

All tests and characterization techniques were performed at room temperature (RT) unless specified.

**1.2 Syntheses**

**1.2.1 Synthesis of amine-modified, amphiphilic polydimethylsilixane (NH_2_-*A*-PDMS)**

NH_2_-*A*-PDMS was synthesized following the procedures given in Scheme S1. The target product (**6**) was obtained as a viscous fluid with a viscosity of 1165 mPa⋅s. The a, b, c values are estimated to be 5, 170 and 9, respectively, based on the molar ratio of the starting materials in Step III. The crude product was directly used for the next step without further purification.

**Scheme S1.** The synthetic route of NH_2_-*A*-PDMS.

**1.2.2 Synthesis of CDs-linked PDMS (CDs-Si)**

CDs-Si was prepared by pyrolysis of CA in the as-prepared NH_2_- *A* -PDMS. Typically, 10 g NH_2_-*A*-PDMS was used in each experiment, to which varying amount of CA was added. Totally seven mixtures were prepared, with the weight ratio of CA to NH_2_-*A*-PDMS of 1:1000, 1:100, 1:50, 1:20, 1:10, 1:5 and 1:1. The mixture were pyrolysised at 200 °C for 2 h. After being cooled to room temperature, each of the sample was dissolved in 200 mL EtOH and subjected to filtration to remove the trace amount of insoluble substance. Finally, EtOH was removed under reduced pressure to afford CDs-Si.

**1.2.3 Synthesis of silicone-modified BODIPY (Si-BODIPY)**

Si-BODIPY was prepared following the procedures given in Scheme S2.

**Scheme S2.** The synthetic route of Si-BODIPY.

**1.2.3.1 Synthesis of 3-(3-chloropropyl)-1,1,1,5,5,5-hexamethyl-3-((trimethylsilyl)oxy)trisiloxane (8)**

The chlorine-terminated, branched silicone **8** was synthesized following the procedures reported in the literature.^1^

**1.2.3.2 Synthesis of 4-(3-(1,1,1,5,5,5-hexamethyl-3-((trimethylsilyl)oxy)trisiloxan-3-yl)propoxy)benzaldehyde (10)**

The silicone-modified benzaldehyde (**10**) was obtained by reacting **8** with 4-hydroxybenzaldehyde (**9**). In brief, **8** (37.3 g, 100 mmol), **9** (6.11 g, 50 mmol), K_2_CO_3_ (34.56 g, 250 mmol) and KI (16.6 g, 100 mmol) were dissolved in 100 mL N, N-dimethylformamide (DMF) in argon atmosphere and stirred for 10 min at room temperature. After that, the mixture was further heated to 110℃ and stirred for 4 h. After being cooled to room temperature, the mixture was extracted with CH_2_Cl_2_ and saturated NaCl aqueous solution three times. The organic phase was combined and dried with Na_2_SO_4_. The organic solvent was removed under reduced pressure. The crude product was purified by column chromatography (silica gel, petroleum ether / dichloromethane = 3: 1) to afford **10** as a colorless oil with a yield of 67.1%. ^1^H NMR (400 MHz, CDCl_3_) δ(ppm) = 9.87 (s, 1H), 7.82 (d, J = 8.8 Hz, 2H), 6.99 (d, J = 8.8 Hz, 2H), 4.00 (t, J = 6.8 Hz, 2H), 1.91-1.79 (m, 2H), 1.34-1.21 (m, 2H), 0.11 (s, 27H). ^13^C NMR (400 MHz, CDCl_3_) δ(ppm): 191, 164.4, 132.13, 129.83, 114.89, 70.57, 23.29, 10.47, 1.88.

**1.2.3.3 Synthesis of Si-BODIPY**

**10** (2.98 g, 6.5 mmol) and 2,4-dimethylpyrrole (**11**, 1.55 g, 16.25 mmol) were dissolved in 120 mL of CH_2_Cl_2_ in an argon atmosphere under stirring. A catalytic amount of TFA was added and the mixture was stirred overnight. Suspension of DDQ (2.95 g, 13 mmol) in CH_2_Cl_2_ (15 mL) was added dropwise to the reaction system, and stirring was continued for 4 h at room temperature. TEA (9.87 g, 97.5 mmol) and BF_3_⋅OEt_2_ (13.84 g, 97.5 mmol) were added dropwise at 0 °C under stirring, after which the mixture was warmed to room temperature and stirring was continued for 4 h. After the reaction was stopped, the mixture was washed with saturated NaHCO_3_ aqueous solution three times. The organic phase was separated and washed three times with saturated NaCl aqueous solution, followed by drying with anhydrous Na_2_SO_4_. The organic solvent was removed under reduced pressure and the crude product was purified by column chromatography (silica gel, petroleum ether/CH_2_Cl_2_ = 3:1) to afford the target compound as an orange solid with a yield of 34.3%. ^1^H NMR (400 MHz, CDCl_3_) δ(ppm) = 7.15 (d, J = 8.7 Hz, 2H), 6.99 (d, J = 8.7 Hz, 2H), 5.97 (s, 2H), 3.97 (t, J = 6.8 Hz, 2H), 2.55 (s, 6H), 1.92-1.80 (m, 2H), 1.43 (s, 6H), 0.65-0.56 (m, 2H), 0.11 (s, 27H); ^13^C NMR (400 MHz, CDCl_3_) δ: 159.83, 155.31, 143.33, 142.17, 132.01, 129.25, 126.88, 121.19, 115.19, 70.27, 23.43, 14.74, 10.52, 2.20, 1.90. MS [M+H] - calcd C_31_H_51_BF_2_N_2_O_4_Si_4_ - 676.9058; found: 677.3100.


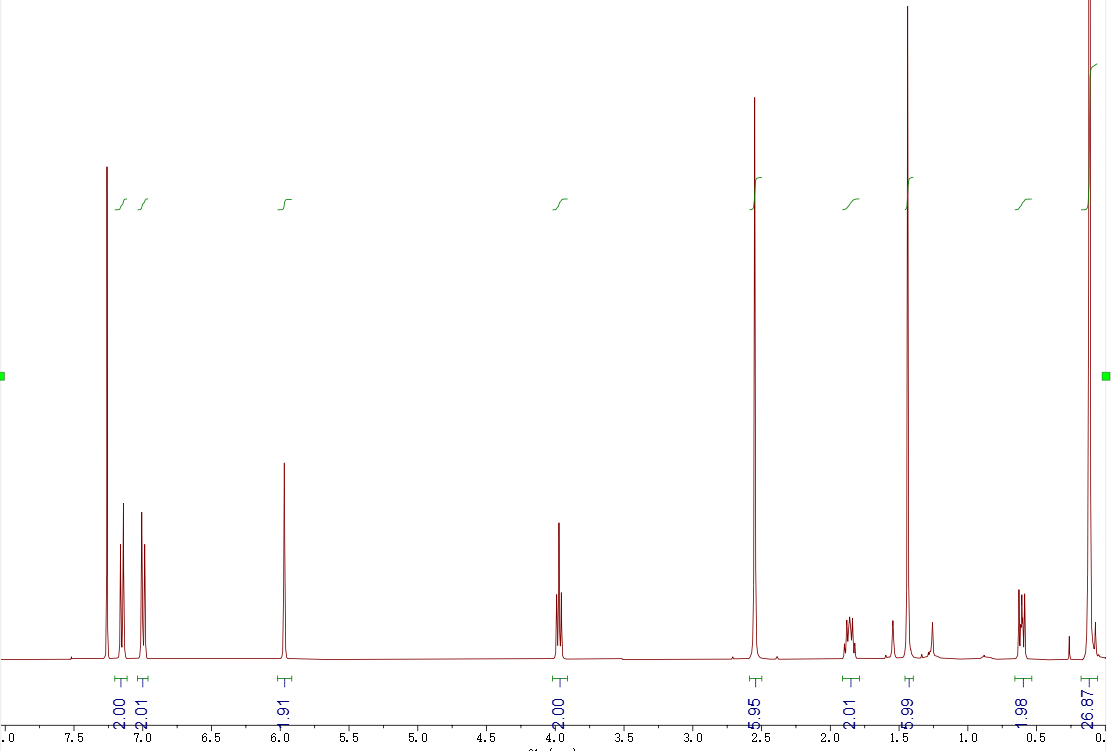


**Scheme S3.** ^1^H NMR spectrum of Si-BODIPY.

**
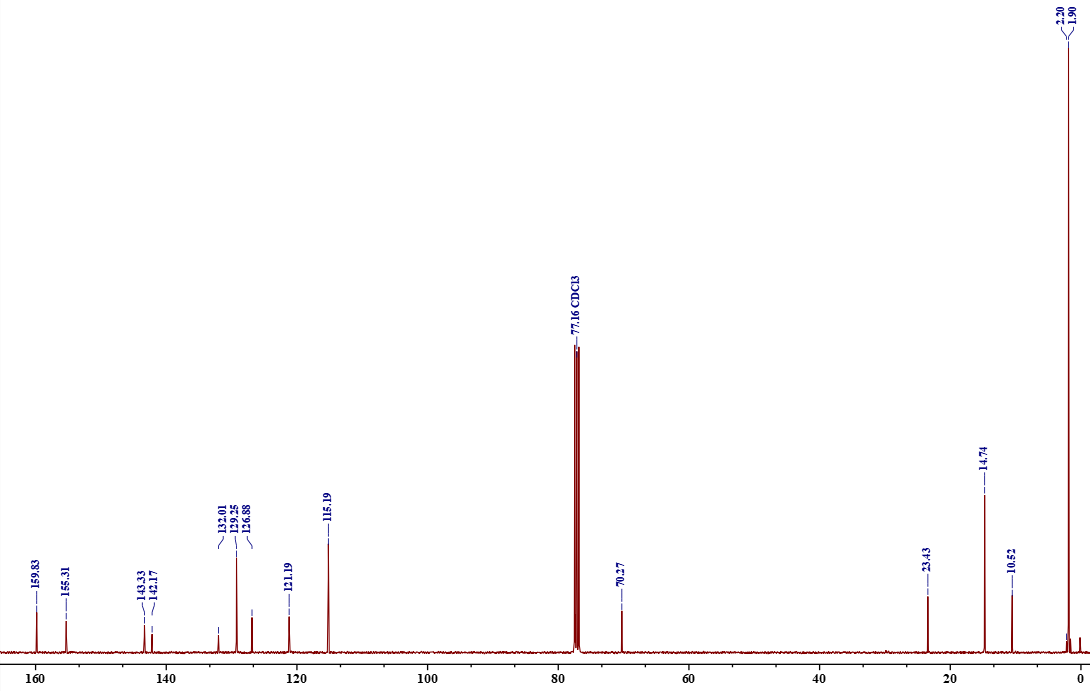
**

**Scheme S4.** ^13^C NMR spectrum of Si-BODIPY.

**2 Calculations of key parameters for FRET**

**2.1 Calculations in CDs-Si/Si-BODIPY and CDs-Si/RhB binary mixtures**

The Fröster radius (R_0_) was calculated by Förster’s Theory.^2^ First, overlap between the emission of the donor and the absorbance (excitation spectrum) of the acceptor was obtained by equation 1:

$J\left( \lambda\right)=\int_{0}^{\infty} F_{D}(\lambda)\varepsilon(\lambda)\lambda^{4}d\lambda$**/**$\int_{0}^{\infty} F_{D}(\lambda)d\lambda$ (1)

where *F*_D_ (λ) is the fluorescence emission spectrum of the donor, whiles *ε* (λ) is the molar absorption coefficient of the acceptor. λ denotes the wavelength of the absorption or emission spectrum. F_D_ (λ) is a dimensionless term in the formula and the value of denominator is normalized to 1 before calculation. With these parameters, *J*(λ) of the three donor/acceptor pairs involved in current study, i.e., CDs-Si/Si-BODIPY, CDs-Si/RhB and Si-BODIPY/RhB, could be calculated to be 4.13×10^14^ M^-1^⋅cm^-1^⋅nm^-4^, 6.65×10^14^ M^-1^⋅cm^-1^⋅nm^-4^ and 2.56×10^15^ M^-1^⋅cm^-1^⋅nm^-4^, respectively.

R_0_ of the energy transfer system can be calculated as follows:

$R_{0}=0.0211\left[ \kappa^{2}n^{-4}\Phi_{D} J\left( \lambda\right) \right]^{1/6}$ (2)

where *n* is the refractive index of the medium, which is 1.3611 in current work (for EtOH). *Φ*_D_ is the quantum yield of the donor (CDs-Si in current work) in the absence of the acceptor, which is 47.51%. *κ* denotes the angle between the emission dipole of the donor and the absorption dipole of the acceptor. For randomly arranged building blocks, a value of 2/3 was normally taken for *κ*^2^. R_0_ of CDs-Si/Si-BODIPY, CDs-Si/RhB and Si-BODIPY/RhB, can then be calculated to be 3.9 nm, 4.2 nm and 5.1 nm, respectively.

Energy transfer efficiency (Φ), is the ability to transfer energy from donor to acceptor, that is, the ratio of the fluorescence intensity of the donor at the optimum emission in the absence (denoted as *I*_D_) and presence (denoted as *I*_DA_) of the acceptor. Calculations of Φ were based on the methods reported in the literatures, which are typical for FRET systems prepared in solution.^3^ It reads:

$=1-I_{\mathrm{DA}}/I_{D}$ (3)

where I_DA_ and I_D_ are the fluorescence intensities of the donor/acceptor pair and the donor alone when excited at a specific wavelength.

For CDs-Si/Si-BODIPY binary mixture, I_DA_ and I_D_ are the fluorescence intensities at 460 nm (included in the superscript of equation 4) of the CDs-Si/Si-BODIPY binary mixture and CDs-Si, respectively, when excited at 390 nm (included in the subscript of equation 4). In this case, Φ is denoted as Φ_12_. Equation 3 can be expressed by:

${{}_{12}=1-I}_{CDs-Si/Si-BODIPY, 390 nm}^{460 nm}/I_{CDs-Si, 390 nm}^{460 nm}$ (4)

Similarly for CDs-Si/RhB binary mixture, I_DA_ and I_D_ are the fluorescence intensities at 460 nm of the CDs-Si/RhB binary mixture and CDs-Si, respectively, when excited at 390 nm. Φ is denoted as Φ_13_. Equation 3 can be expressed by:

${{}_{13}=1-I}_{CDs-Si/RhB,390 nm}^{460 nm}/I_{CDs-Si,390 nm}^{460 nm}$ (5)

According to Förster’s Theory,^2^ Φ is related to the rate constants of Förster resonance energy transfer (*k*_ET_), the radiative (*k*_r_) and non-radiative (*k*_nr_) decay constants of donor by the following equation:

*Φ* = *k*_ET_/(*k*_r_ + *k*_nr_ + *k*_ET_) (6)

The sum of *k*_r_ and *k*_nr_ is equal to the reciprocal of the fluorescence lifetime of the donor (*τ*_D_) in the absence of acceptor, which is 11.4 ns. The *k*_ET_ of the donor/acceptor combinations in CDs-Si/Si-BODIPY and CDs-Si/RhB binary mixtures at varying concentration of the acceptor could then be calculated, which are summarized in Tables S2 and S4.

The averaged distance between the donor and the acceptor (*d*) is related to *k*_ET_ by the following equation:

*k*_ET_ = (1/*τ*_D_)×(R_0_/d)^6^ (7)

The values of the two ternary mixtures (CDs-Si/Si-BODIPY and CDs-Si/RhB) were obtained and also summarized in Tables S2 and S4.

Antenna effect (AE), is the ability of the acceptor to harvest energy from the donor. It is commonly expressed as:

$AE=(I_{DA, 390}-I_{D, 390})/I_{DA, {}_{2}}$ (8)

For CDs-Si/Si-BODIPY binary mixture, equation 8 can be written as:

${{AE}_{12}=(I}_{CDs-Si/Si-BODIPY,390 nm}^{{}_{em} of Si-BODIPY}-I_{CDs-Si,390 nm}^{{}_{em} of Si-BODIPY})/I_{CDs-Si/Si-BODIPY,460 nm}^{{}_{em} of Si-BODIPY}$ (9)

where $I_{CDs-Si/Si-BODIPY,390 nm}^{{}_{em} of Si-BODIPY}$is the fluorescence intensity of Si-BODIPY in the binary mixture at its optimum emission when excited at 390 nm, which shows slight bathochromic shift (from 512nm to 520 nm, see Figure S15b) with increasing *c*_Si-BODIPY_. $I_{CDs-Si,390 nm}^{{}_{em} of Si-BODIPY}$ is the fluorescence intensity of CDs-Si when existing alone under excitation of 390 nm at the same wavelength of the optimum emission of Si-BODIPY in the binary mixture. At each *c*_Si-BODIPY_, the curve of CDs-Si was normalized with that of the binary mixture at 460 nm before data were extracted for calculation. $I_{CDs-Si/Si-BODIPY,460 nm}^{{}_{em} of Si-BODIPY}$ is the fluorescence intensity of Si-BODIPY in the binary mixture at its optimum emission when excited at 460 nm (see Figure S18a for the raw data).

For CDs-Si/RhB binary mixture, equation 8 can be written as:

${{AE}_{13}=(I}_{CDs-Si/RhB,390 nm}^{{}_{em} of RhB}-I_{CDs-Si,390 nm}^{{}_{em} of RhB})/I_{CDs-Si/RhB,490 nm}^{{}_{em} of RhB}$ (10)

where $I_{CDs-Si/RhB,390 nm}^{{}_{em} of RhB}$ is the fluorescence intensity of RhB in the binary mixture at its optimum emission when excited at 390 nm, which shifts gradually from 573 nm to 584 nm with increasing *c*_RhB_ (see Figure 2d in the maintext). $I_{CDs-Si,390 nm}^{{}_{em} of RhB}$ is the fluorescence intensity of CDs-Si when existing alone under excitation at 390 nm at the same wavelength of the optimum emission of RhB in the binary mixture. At each *c*_RhB_, the curve of CDs-Si was normalized with that of the binary mixture at 460 nm before data were extracted for calculation. $I_{CDs-Si/RhB,490 nm}^{{}_{em} of RhB}$ is the fluorescence intensity of RhB in the binary mixture at its optimum emission when excited at 490 nm (the curve is given in Figure S21).

**2.2 Calculations in CDs-Si/Si-BODIPY/RhB ternary mixtures**

As demonstrated in the maintext, in CDs-Si/Si-BODIPY/RhB ternary mixture, sequential FRET from CDs-Si to Si-BODIPY and further to RhB occurs, together with a parallel FRET from CDs-Si directly to RhB. These two processes are competitive, which would influence Φ. In this case, Φ from CDs-Si to Si-BODIPY is defined as Φ_12_′, and Φ from CDs-Si to RhB is defined as Φ_13_′. They are expressed by equations 11 and 12:

*Φ*_12_′ = *k*_ET(12)_/(*k*_r(12)_ + *k*_nr(12)_ + *k*_ET(12)_ + *k*_ET(13)_) (11)

*Φ*_13_′ = *k*_ET(13)_/(*k*_r(13)_ + *k*_nr(13)_ + *k*_ET(13)_ + *k*_ET(12)_) (12)

where the subscripts _(12)_ and _(13)_ denote parameters obtained from CDs-Si/Si-BODIPY and CDs-Si/RhB, respectively, which are included in Table S2 and Table S4 respectively.

The average distance between CDs-Si/Si-BODIPY (*d*_12_′) and CDs-Si/RhB (*d*_13_′) is obtained by equation 12 and 13:

$d_{12}={R_{0(12)}[(1-{}_{12}-{}_{13} )/{}_{12} ]}^{1/6}$ (13)

$d_{13}={R_{0(13)}[(1-{}_{13}-{}_{12} )/{}_{13} ]}^{1/6}$ (14)

where *R*_0(12)_ and *R*_0(13)_ are Fröster radius calculated by equation 2 in CDs-Si/Si-BODIPY and CDs-Si/RhB binary mixtures, which is 3.91 nm and 4.24 nm, respectively.

There is an additional FRET from Si-BODIPY to RhB in the ternary mixture, whose Φ is defined as Φ_23_′, which is given by:

${{}_{23}=1-I}_{CDs-Si/Si-BODIPY/RhB,390 nm}^{515 nm}/I_{CDs-Si/Si-BODIPY,390 nm}^{515 nm}$ (15)

where $I_{CDs-Si/Si-BODIPY/RhB,390 nm}^{515 nm}$ and $I_{CDs-Si/Si-BODIPY,390 nm}^{515 nm}$ are the fluorescence intensities at 515 nm of the ternary mixture and CDs-Si/Si-BODIPY binary mixture, respectively, when excited at 390 nm.

The AE (AE_23_′) was obtained following equation 16:

${{AE}_{23}=(I}_{CDs-Si/Si-Bodipy/RhB,390 nm}^{{}_{em} of RhB}-I_{CDs-Si/Si-BODIPY,390 nm}^{{}_{em} of RhB})/I_{CDs-Si/Si-BODIPY/RhB,490 nm}^{{}_{em} of RhB}$ (16)

where $I_{CDs-Si/Si-Bodipy/RhB,390 nm}^{{}_{em} of RhB}$ is the fluorescence intensity of RhB in the ternary mixture at its optimum emission when excited at 390 nm, which shifts gradually from 569 nm to 585 nm with increasing *c*_RhB_ (see Figure 2f in the maintext). $I_{CDs-Si/Si-BIDIPY,390 nm}^{{}_{em} of RhB}$ is the fluorescence intensity of the CDs-Si/Si-BODIPAY binary mixture when excited at 390 nm, recorded at the same series of wavelengths of the optimum emission of RhB in the ternary mixture. At each *c*_RhB_, the curve of the CDs-Si/Si-BODIPAY binary mixture was normalized with that of the ternary mixture at 515 nm before data were extracted for calculation. $I_{CDs-Si/Si-BODIPY/RhB,490 nm}^{{}_{em} of RhB}$ is the fluorescence intensity of RhB in the ternary mixture when excited at 490 nm (the curve is given in Figure S24).

The *k*_ET_ of this additional FRET is still calculated with equation 6, but the sum of *k*_r_ and *k*_nr_ is in this case equal to the reciprocal of the fluorescence lifetime of Si-BODIPY when embedded in the CDs-Si/Si-BODIPY binary mixture, which is 7.1 ns (see Figure 2g in the maintext).

The overall Φ is defined as *Φ*_overall_ = *Φ*_123_ + *Φ*_13_′ = *Φ*_23_′ × *Φ*_12_′ + *Φ*_13_′

The calcuated parameters for the two ternary mixtures are given in Table S6-S8.

**2.3 Calculations in DAE-containing systems**

Under 254 nm UV irradiation, O-DAE could not be converted to C-DAE in one hundred percent. For this reason, the molar absorption coefficient of C-DAE is unavailable. But calculations of *Φ* and *k*_ET_ is still possible in CDs-Si/C-DAE binary mixture and CDs-Si/Si-BODIPY/C-DAE ternary mixture.

The *Φ* of energy transfer from CDs-Si to C-DAE in the binary mixture (denoted as *Φ*_14_) is obtained by:

${{}_{14}=1-I}_{t, 390 nm}^{460 nm}/I_{0, 390 nm}^{460 nm}$ (17)

where $I_{t, 390 nm}^{460 nm}$ and $I_{0, 390 nm}^{460 nm}$ are fluorescence intensity at 460 nm of the mixture at time *t* and time 0, respectively, when excited at 390 nm.

The rate constant of the energy transfer (*k*_ET(14)_) could then be obtained by equation 6.

In CDs-Si/Si-BODIPY/C-DAE ternary mixture, *Φ* of energy transfer from Si-BODIPY to C-DAE (*Φ*_24_′) is obtained by:

${{}_{24}=1-I}_{t, 390 nm}^{515 nm}/I_{0, 390 nm}^{515 nm}$ (17)

where $I_{t, 390 nm}^{515 nm}$ and $I_{0, 390 nm}^{515 nm}$ are fluorescence intensity at 515 nm of the mixture at time *t* and time 0, respectively, when excited at 390 nm.

The rate constant of the energy transfer (*k*_ET(24)_) could then be obtained by equation 6.

*Φ* of energy transfer from CDs-Si to Si-BODIPY (*Φ*_12_′′) and from CDs-Si to C-DAE (*Φ*_14_′) could be obtained by equations 18 and 19:

*Φ*_12_′′ = *k*_ET(12)_/(*k*_r(12)_ + *k*_nr(12)_ + *k*_ET(12)_ + *k*_ET(14)_) (18)

*Φ*_14_′ = *k*_ET(14)_/(*k*_r(14)_ + *k*_nr(14)_ + *k*_ET(14)_ + *k*_ET(12)_) (19)

The overall Φ is defined as *Φ*_overall_ = *Φ*_124_ + *Φ*_14_′ = *Φ*_24_′ × *Φ*_12_′ + *Φ*_14_′

The calcuated parameters are given in Table S9.

As we demonstrated in the maintext, the CDs-Si/Si-BODIPY/RhB/C-DAE quaternary system is too complicated, on which no calculations were made.

**3. Additional data and graph**

**Figure S1.** FTIR spectra of CDs-Si obtained at different *r* as indicated inset. The spectrum of NH_2_-A-PDMS is also given for comparison. a) The whole spectra in the range of 4000-400 cm^-1^. b) Magnified spectra in the range of 1800-1500 cm^-1^.

**Figure S2.** UV-vis absorption of NH_2_-*A*-PDMS in EtOH (1 mg⋅mL^-1^).

**Figure S3.** Emission of CDs-Si in EtOH as a function of concentration. Recorded at λ_ex_ = 390 nm. a) The emission curves. b) Variation of the PL intensity with concentration.

**Figure S4.** Variation of surface tension as a function of the concentration of NH_2_-*A*-PDMS in EtOH. The surface tension of EtOH without NH_2_-*A*-PDMS is indicated by the dashed line.


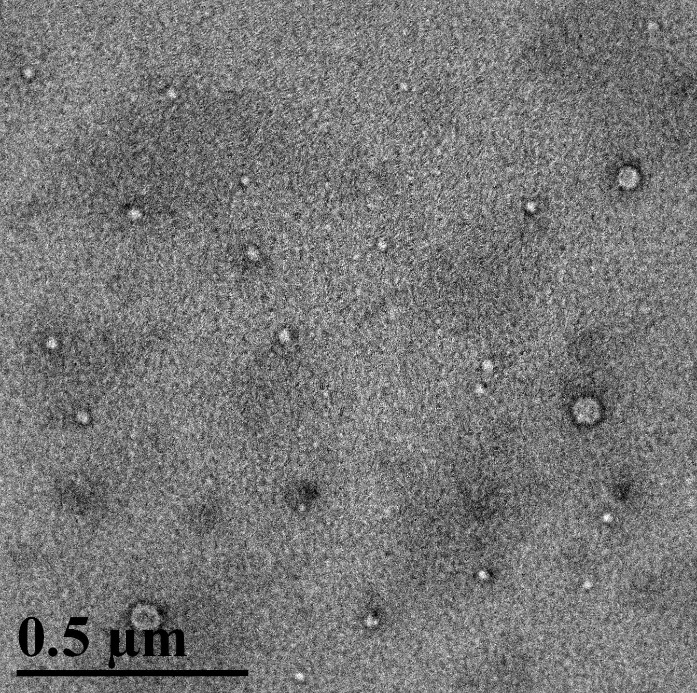


**Figure S5.** A typical TEM image showing the formation of capsules from NH_2_-*A*-PDMS in EtOH at a concentration of 0.5 mg⋅mL^-1^.

**Figure S6.** Comparison of the sizes of the capsules from NH_2_-A-PDMS and CDs-Si in EtOH. The concentration is fixed at 0.2 mg⋅mL^-1^.

**Figure S7.** Size distribution from DLS measurement for CDs-Si in EtOH (0.2 mg⋅mL^-1^).

**Figure S8.** TEM images of CDs-Si in EtOH with a concentration of 0.02 mg⋅mL^-1^  (a) and 2.0 mg⋅mL^-1^ (b), respectively. The two images were handled with the same magnification for better comparison (scale bar: 500 nm).

**Figure S9.** SEM images with different magnifications from a 2 mg⋅mL^-1^ EtOH solution of CDs-Si.

**Figure S10.** a) Emission of Si-BODIPY in EtOH (100 μmol⋅L^-1^), recorded at varying λ_ex_. b) Statistics of the PL intensity at λ_em_ = 520 nm at different λ_ex_.

**Figure S11.** Emission of Si-BODIPY in EtOH at different concentration as indicated, recorded at λ_ex_ of 390 nm (a) and 460 nm (b) respectively.

**Figure S12.** PL decay of Si-BODIPY in EtOH, recorded with a concentration of 90 μmol⋅L^-1^.

**Figure S13.** a) Emission of RhB in EtOH (50 μmol⋅L^-1^), recorded at varying λ_ex_. b) Statistics of the PL intensity at λ_em_ = 580 nm at different λ_ex_.

**Figure S14.** Emission of different concentrations of RhB in EtOH as indicated, recorded at λ_ex_ of 390 nm (a), 490 nm (b) and 520 nm (c), respectively.

**Figure S15.** a) Magnified graph highlighting the emission in the green light region during the titration of CDs-Si in EtOH (2 mg⋅mL^-1^) with Si-BODIPY, λ_ex_ = 390 nm. b) Statistics of the position of the emission (in terms of λ_em_) of the curves in graph a. c) Statistics of the PL intensity of the curves in graph a. For comparison, data recorded at 390 nm and 460 nm for Si-BODIPY alone in EtOH were also given (the original emission curves could be found in Figure S10).

Data notion: Both the PL intensity and the variation of this peak with *c*_Si-BODIPY_ in the binary mixture is different from those of Si-BODIPY alone in EtOH when excited at 390 nm, indicating the occurring of FRET.

**Figure S16.** a) Normalized emission in the blue light region during the titration of CDs-Si in EtOH (2 mg⋅mL^-1^) with Si-BODIPY, λ_ex_ = 390 nm. b) Statistics of the PL intensity and the extent of the hypochromatic shift of the curves in graph a.

**Figure S17.** Normalized emission of CDs-Si and the absorption of Si-BODIPY. The overlapped area is highlighted.

**Figure S18.** a) Emission curves of the samples during titration of a CDs-Si solution in EtOH (2 mg⋅mL^-1^) with Si-BODIPY, λ_ex_ = 460 nm. b) Plot of I_DA,390_, I_D,390_ and I_DA,460_. These data are needed for the calculation of AE.

**Figure S19.** Normalized emission of CDs-Si (λ_ex_ = 390 nm) and the absorption of RhB. The overlapped area is highlighted.

**Figure S20.** PL decay recorded at λ_em_ = 460 nm (λ_ex_ = 390 nm) for CDs-Si in EtOH (2 mg⋅mL^-1^) during titration with RhB. Values of the averaged lifetimes are included.

**Figure S21.** a) Emission curves of the samples during titration of a CDs-Si solution in EtOH (2 mg⋅mL^-1^) with RhB, λ_ex_ = 490 nm. b) Plot of I_DA,390_, I_D,390_ and I_DA,490_. These data are needed for the calculation of AE.

**Figure S22.** a) Variation of the emission of the sample containing 2 mg⋅mL^-1^ CDs-Si and 90 μmol⋅L^-1^ Si-BODIPY upon the addition of RhB.

**Figure S23.** Plot of key parameters involved in the FRET from Si-BODIPY to RhB in the ternary mixture containing 2 mg⋅mL^-1^ CDs-Si, 50 μmol⋅L^-1^ Si-BODIPY and varying concentration of RhB (The titration curves could be found in Figure S22).

**Figure S24.** Emission curves (a, c) and plot of I_DA,390_, I_D,390_ and I_DA,490_ (b, d) of the samples during titration of a CDs-Si (2 mg⋅mL^-1^)/Si-BODIPY mixture in EtOH with RhB, λ_ex_ = 490 nm. The concentration of Si-BODIPY is 90 (a, b) and 50 (c, d) μmol⋅L^-1^, respectively. These data are needed for the calculation of AE.

**Figure S25.** Typical TEM images (a) and the statistics of the diameters (b) of the capsules for the binary mixture of 2.0 mg⋅mL^-1^ CDs-Si/90 μmol⋅L^-1^ Si-BODIPY in EtOH. c,d) Results for the mixture after diluted 10 times.

**Figure S26.** A typical TEM image (a) and the statistics of the diameters (b) of the capsules for the ternary mixture of 2.0 mg⋅mL^-1^ CDs-Si/90 μmol⋅L^-1^ Si-BODIPY/50 μmol⋅L^-1^ RhB in EtOH.

**Figure S27.** SEM mapping of the film prepared from an EtOH solution of 2 mg⋅mL^-1^ CDs-Si and 90 μmol⋅L^-1^ Si-BODIPY.

**Figure S28.** SEM mapping of the film prepared from an EtOH solution of 2 mg⋅mL^-1^ CDs-Si, 90 μmol⋅L^-1^ Si-BODIPY and 50 μmol⋅L^-1^ RhB.

**Figure S29.** Variation of zeta potential of two series samples during titration. Squares: Titration of CDs-Si (2 mg⋅mL^-1^) with Si-BODIPY. Circles: Titration of CDs-Si (2 mg⋅mL^-1^)/Si-BODIPY (90 μmol⋅L^-1^) with RhB.

**Data Notion:** i) For zeta potential obtained in nonaqueous solution (EtOH in this case), the explicit explanation might be problematic. However, the change of the value induced by titration unambiguously proved the role played by the electrostatic interaction during the self-assembly.

ii) For CDs prepared from CA, a negatively-charge surface is expected due to the presence of carboxy or hydroxy groups. However, we noticed that the capsules formed by CDs-Si are positively charged, which basically indicated that the amine groups from NH_2_-*A*-PDMS were in excess during the pyrolysis.

**Figure S30.** a) Illustration of the preparation of the blue-emission CDs from CA and TEPA. b) The emission of TEPA-CDs in EtOH with 365 nm excitation. Other details of the characterization will be published independently. c-e) Evidences of the absence of FRET between TEPA-CDs and Si-BODIPY. Samples were excited at 365 nm unless otherwise stated. c) Variation of the emission of Si-BODIPY (100 μmol·L^-1^) upon the addition of TEPA-CDs. (d) PL decay recorded at λ_em_ = 460 nm for TEPA-CDs (0.1 mg·mL^-1^) without and with 100 μmol·L^-1^ Si-BODIPY. (e) PL decay recorded at λ_em_ = 520 nm for Si-BODIPY(100 μmol·L^-1^) without and with 0.1 mg·mL^-1^ TEPA-CDs.

**Figure S31.** Statistics of the intensity of the two peaks of DAE upon alternative irradiation of UV and visible light (up to ten cycles). The absorption curves have been given in Figure 5b in the maintext.

**Figure S32.** PL decay recorded at λ_em_ = 460 nm (λ_ex_ = 390 nm) for the binary mixture containing 2 mg⋅mL^-1^ CDs-Si and 200 μmol⋅L^-1^ DAE at 0 and 9 min. Values of <τ> are included.

**Figure S33.** Decreasing of the PL intensity for the two emission peaks at 460 nm and 520 nm (λ_ex_ = 390 nm) under continuous UV irradiation for the ternary mixture containing 2 mg⋅mL^-1^ CDs-Si, 90 μmol⋅L^-1^ Si-BODIPY and 200 μmol⋅L^-1^ DAE.

**Figure S34.** PL decay recorded at λ_em_ = 520 nm (λ_ex_ = 390 nm) for the ternary mixture containing 2 mg⋅mL^-1^ CDs-Si, 90 μmol⋅L^-1^ Si-BODIPY and 200 μmol⋅L^-1^ DAE at three selected time as indicated. Values of <τ> are included.

**Figure S35.** Decreasing of the PL intensity for the three emission peaks at 460 nm, 520 nm and 580 nm (λ_ex_ = 390 nm) under continuous UV irradiation for the quaternary mixture containing 2 mg⋅mL^-1^ CDs-Si, 90 μmol⋅L^-1^ Si-BODIPY, 50 μmol⋅L^-1^ RhB and 200 μmol⋅L^-1^ DAE.

**Figure S36.** a) Variations of the emission of the sample containing 2 mg⋅mL^-1^ CDs-Si, 90 μmol⋅L^-1^ Si-BODIPY, 50 μmol⋅L^-1^ RhB and 200 μmol⋅L^-1^ DAE under continuous irradiation of 254 UV light. b) Decreasing of the PL intensity for the three emission peaks in a.

**Figure S37.** Emission curves upon alternative irradiation of UV and visible light (up to ten cycles) for a) the ternary mixture containing 2 mg⋅mL^-1^ CDs-Si, 90 μmol⋅L^-1^ Si-BODIPY and 200 μmol⋅L^-1^ DAE, and the quaternary mixture containing 2 mg⋅mL^-1^ CDs-Si, 90 μmol⋅L^-1^ Si-BODIPY, 50 μmol⋅L^-1^ RhB and 200 μmol⋅L^-1^ DAE.

**4. Tables**

**4.1 Details of lifetime measurements**

**Table S1.** Parameters of each series of samples during the time-resolved fluorescence measurements, recorded in EtOH at λ_ex_ = 390 nm.

|  | | τ_1_ | τ_2_ | χ | <τ> |
| --- | --- | --- | --- | --- | --- |
| CDs-Si  (2 mg⋅mL^-1^) | / | 11.4 |  | 1.1 | Figure 2c |
|  | + 90 μmol⋅L^-1^ Si-BODIPY | 1.87 | 10.7 | 1.0 |  |
|  | + 25 μmol⋅L^-1^ RhB | 3.6 | 11.7 | 0.9 | Figure S20 |
|  | + 50 μmol⋅L^-1^ RhB | 2.6 | 11.3 | 1.0 |  |
|  | + 200 μmol⋅L^-1^ DAE, 254 nm UV for 9 min | 1.65 | 11.36 | 1.1 | Figure S29 |
| Si-BODIPY (90 μmol⋅L^-1^) | | 3.2 | 11.2 | 1.0 | Figure S12 |
| CDs-Si (2 mg⋅mL^-1^)/ Si-BODIPY (90 μmol⋅L^-1^) | / | 5.0 | 10.2 | 1.1 | Figure 2g |
|  | + 12 μmol⋅L^-1^ RhB | 4.7 | 10.5 | 1.0 |  |
|  | + 35 μmol⋅L^-1^ RhB | 4.3 | 10.5 | 1.0 |  |
|  | + 200 μmol⋅L^-1^ DAE, 254 nm UV for 4 min | 4.0 | 9.3 | 1.1 | Figure S31 |
|  | + 200 μmol⋅L^-1^ DAE, 254 nm UV for 9 min | 3.9 | 9.3 | 1.0 |  |

**4.2 Parameters during FRET process for CDs-Si/Si-BODIPY binary mixture**

**Table S2.** Variations of k_ET_, the sum of radiative and nonradiative rate constants (k_r_ + k_nr_), the efficiency of energy transfer (Φ_12_) and the distance between CDs-Si and Si-BODIPY (*d*_12_). The concentration of CDs-Si is fixed at 2 mg⋅mL^-1^.

| *c*_Si-BODIPY_ (μmol⋅L^-1^) | *k*_ET_ (10^7^ s^-1^) | *k*_r_ + *k*_nr_ (10^7^ s^-1^) | Φ_12_ (%) | *d*_12_ (nm) |
| --- | --- | --- | --- | --- |
| 10 | 1.8 | 8.7 | 16.8 | 5.09 |
| 20 | 5.5 | 8.7 | 38.6 | 4.22 |
| 30 | 8.8 | 8.7 | 50.1 | 3.90 |
| 40 | 14 | 8.7 | 62.1 | 3.62 |
| 50 | 19 | 8.7 | 68.9 | 3.44 |
| 60 | 31 | 8.7 | 78.1 | 3.17 |
| 70 | 39 | 8.7 | 81.8 | 3.05 |
| 80 | 54 | 8.7 | 86 | 2.89 |
| 90 | 75 | 8.7 | 89.5 | 2.73 |

**Table S3.** AE_12_ and the raw data adopted during the calculation.

| *c*_Si-BODIPY_ (μmol⋅L^-1^) | I_DA_, 390 | I_D_, 390 | I_DA_, 460 | AE_12_ |
| --- | --- | --- | --- | --- |
| 0 | 154478 | 284640 |  |  |
| 10 | 302749 | 236821 | 676619 | 0.10 |
| 20 | 402320 | 174900 | 993945 | 0.23 |
| 30 | 437880 | 142107 | 1104837 | 0.27 |
| 40 | 466330 | 108007 | 1214438 | 0.30 |
| 50 | 472202 | 88419 | 1134581 | 0.34 |
| 60 | 463557 | 62460 | 1119773 | 0.36 |
| 70 | 455505 | 51092 | 941119 | 0.43 |
| 80 | 446673 | 39795 | 843865 | 0.48 |
| 90 | 436922 | 29949 | 738713 | 0.55 |

**4.3 Parameters during FRET process for CDs-Si/RhB binary mixture**

**Table S4.** Variations of k_ET_, the sum of radiative and nonradiative rate constants (k_r_ + k_nr_), the efficiency of energy transfer (Φ_13_) and the distance between CDs-Si and RhB (d_13_). The concentration of CDs-Si is fixed at 2 mg⋅mL^-1^.

| *C*_RhB_ (μmol⋅L^-1^) | k_ET_ (10^7^ s^-1^) | k_r_ + k_nr_ (10^7^ s^-1^) | Φ_13_ (%) | d_13_ (nm) |
| --- | --- | --- | --- | --- |
| 10 | 1.8 | 8.7 | 17.5 | 5.09 |
| 20 | 2.6 | 8.7 | 23.3 | 4.79 |
| 30 | 2.9 | 8.7 | 25 | 4.70 |
| 40 | 3.4 | 8.7 | 27.7 | 4.58 |
| 50 | 4.1 | 8.7 | 31.7 | 4.44 |

**Table S5.** AE_13_ and the raw data adopted during the calculation.

| *c*_Si-BODIPY_ (μmol⋅L^-1^) | I_DA_, 390 | I_D_, 390 | I_DA_, 460 | AE_13_ |
| --- | --- | --- | --- | --- |
| 0 | 52932 | 52932 |  |  |
| 10 | 299393 | 43682 | 1063232 | 0.24 |
| 20 | 416881 | 40590 | 1588127 | 0.24 |
| 30 | 490809 | 39684 | 1878167 | 0.24 |
| 40 | 608303 | 38255 | 2157488 | 0.26 |
| 50 | 648154 | 36160 | 2225630 | 0.27 |

**4.4 Parameters during FRET process for the ternary mixture**

**Table S6.** Details of the FRET from Si-BODIPY to RhB in the mixture of CDs-Si (2 mg⋅mL^-1^)/Si-BODIPY (90 μmol⋅L^-1^) upon the addition of RhB.

| *c*_RhB_  (μmol⋅L^-1^) | k_ET_  (10^8^ s^-1^) | k_r_ + k_nr_  (10^8^ s^-1^) | Φ_23_′ (%) | I_DA_, 390 | I_D_, 390 | I_DA_, 490 | AE_23_′ |
| --- | --- | --- | --- | --- | --- | --- | --- |
| 0 |  |  |  | 47604 | 47604 |  |  |
| 5 | 0.53 | 1.42 | 27.4 | 149178 | 34555 | 83871 | 1.37 |
| 10 | 1.21 | 1.42 | 46.2 | 193564 | 25620 | 97207 | 1.72 |
| 15 | 2.15 | 1.42 | 60.3 | 215868 | 18882 | 88761 | 2.22 |
| 20 | 3.48 | 1.42 | 71.1 | 222705 | 13772 | 82765 | 2.52 |
| 25 | 5.32 | 1.42 | 79 | 229525 | 9976 | 72629 | 3.02 |
| 30 | 7.27 | 1.42 | 83.7 | 229774 | 7767 | 63853 | 3.48 |
| 35 | 10.79 | 1.42 | 88.4 | 236714 | 5519 | 58800 | 3.93 |
| 40 | 14.15 | 1.42 | 90.9 | 237040 | 4322 | 53563 | 4.34 |
| 45 | 19.11 | 1.42 | 93.1 | 243679 | 3271 | 47166 | 5.09 |
| 50 | 25.82 | 1.42 | 94.8 | 236788 | 2475 | 45427 | 5.15 |

**Table S7.** Details of the FRET from Si-BODIPY to RhB in the mixture of CDs-Si (2 mg⋅mL^-1^)/Si-BODIPY (50 μmol⋅L^-1^) upon the addition of RhB.

| *c*_RhB_  (μmol⋅L^-1^) | k_ET_  (10^8^ s^-1^) | k_r_ + k_nr_  (10^8^ s^-1^) | Φ_23_′ (%) | I_DA_, 390 | I_D_, 390 | I_DA_, 490 | AE_23_′ |
| --- | --- | --- | --- | --- | --- | --- | --- |
| 0 |  |  |  | 493675 | 493675 |  |  |
| 5 | 0.33 | 1.41 | 19.2 | 1625951 | 399125 | 1033977 | 1.19 |
| 9.8 | 0.71 | 1.41 | 33.4 | 2008146 | 328565 | 1098216 | 1.53 |
| 14.6 | 1.10 | 1.41 | 43.8 | 2300634 | 277607 | 934578 | 2.16 |
| 19.2 | 1.52 | 1.41 | 51..9 | 2500892 | 237658 | 825345 | 2.74 |
| 23.8 | 1.91 | 1.41 | 57.6 | 2669532 | 209563 | 705260 | 3.49 |
| 32.7 | 2.76 | 1.41 | 66.2 | 2655146 | 166638 | 552596 | 4.50 |
| 41.3 | 5.08 | 1.41 | 78.3 | 2966822 | 106973 | 472159 | 6.06 |
| 49.5 | 7.62 | 1.41 | 82.4 | 3144017 | 86715 | 416039 | 7.35 |
| 57.5 | 9.10 | 1.41 | 86.6 | 3206779 | 66378 | 378873 | 8.29 |
| 65.2 | 12.40 | 1.41 | 89.8 | 3306636 | 50318 | 350792 | 9.28 |
| 72.6 | 17.62 | 1.41 | 92.6 | 3340890 | 36728 | 329225 | 10.04 |
| 87 | 21.31 | 1.41 | 93.8 | 3482967 | 30893 | 295813 | 11.67 |

**Table S8.** Parameters of FRET from CDs-Si to Si-BODIPY and from CDs-Si to RhB in the ternary mixture containing 2 mg⋅mL^-1^ CDs-Si, 90 μmol⋅L^-1^ Si-BODIPY and varying concentration of RhB. The overall Φ (Φ_overall_ = Φ_123_ + Φ_13_′ =Φ_23_′ × Φ_12_′ + Φ_13_′) is also included.

| *c*_RhB_  (μmol⋅L^-1^) | Φ_12_′′ (%) | Φ_13_′ (%) | Φ_overall_ (%) | d_12_′ (nm)*^a^* | d_13_′ (nm) |
| --- | --- | --- | --- | --- | --- |
| 0 | 89.5 |  |  | 2.73 |  |
| 10 | 87.7 | 2.1 | 42.6 | 2.73 | 5.52 |
| 20 | 86.9 | 3.0 | 64.8 | 2.73 | 5.19 |
| 30 | 86.6 | 3.3 | 75.8 | 2.73 | 5.10 |
| 40 | 86.1 | 3.9 | 82.2 | 2.73 | 4.96 |
| 50 | 85.4 | 4.7 | 85.7 | 2.73 | 4.80 |

*^a^* With increasing *c*_RhB_, d_12_′ also decreases, but with a very small extent which could not be reflected by the numbers shown here (the change starts at the third decimal place).

**Table S9.** Parameters of FRET involved in CDs-Si/C-DAE and CDs-Si/Si-BODIPY/C-DAE systems. The concentration of CDs-Si, Si-BODIPY and C-DAE is fixed at 2 mg⋅mL^-1^, 90 μmol⋅L^-1^ and 200 μmol⋅L^-1^, respectively.

| *t* (min) | binary mixture | | ternary mixture | | | |
| --- | --- | --- | --- | --- | --- | --- |
|  | k_ET(14)_ (10^7^ s^-1^) | Φ_14_ (%) | k_ET(24)_ (10^7^ s^-1^) | Φ_24_′ (nm) | Φ_12_′ (nm)*^a^* | Φ_14_′ (nm) |
| 1 | 5.22 | 37.3 | 4.16 | 22.8 | 84.34 | 5.87 |
| 2 | 13.89 | 61.3 | 9.82 | 41.1 | 76.85 | 14.23 |
| 3 | 20.76 | 70.3 | 16.80 | 54.4 | 71.80 | 19.87 |
| 4 | 25.76 | 74.6 | 22.50 | 61.5 | 68.52 | 23.53 |
| 5 | 35.76 | 80.3 | 33.02 | 70.1 | 62.78 | 29.93 |
| 6 | 36.92 | 80.8 | 45.34 | 76.3 | 62.18 | 30.61 |
| 7 | 41.64 | 82.6 | 59.27 | 80.8 | 59.84 | 33.22 |
| 8 | 45.38 | 83.8 | 66.40 | 82.5 | 58.10 | 35.16 |
| 9 | 50.50 | 85.2 | 108.39 | 88.5 | 55.89 | 37.63 |

**References**

[1] Y. Huang, L. Meng, M. Guo, P. Zhao, H. Zhang, S. Chen, J. Zhang, S. Feng, *Langmuir* **2018**, *34*, 4382-4389.

[2] J. R. Lockwicz, *Principles of Fluorescence Spectroscopy, 3^rd^ edition,* Springer, **2006**.

[3] W. R. Algar, N. Hildebrandt, S. S. Vogel, I. L. Medintz, *Nature Methods* **2019**, *16*, 815-829; S. Kundu, A. Patra, *Chemical Reviews* **2017**, *117*, 712-757; L. Wu, C. Huang, B. P. Emery, A. C. Sedgwick, S. D. Bull, X.-P. He, H. Tian, J. Yoon, J. L. Sessler, T. D. James, *Chemical Society Reviews* **2020**, *49*, 5110-5139.

1. *Author to whom correspondence should be addressed, E-mail: hgli@sdu.edu.cn.

   Phone: +86-531-88363963. Fax: +86-531-88564750 [↑](#footnote-ref-1)
